# AOP key event relationship report: Linking androgen receptor antagonism with nipple retention

**DOI:** 10.1016/j.crtox.2022.100085

**Published:** 2022-08-30

**Authors:** Emilie Bak Pedersen, Sofie Christiansen, Terje Svingen

**Affiliations:** National Food Institute, Technical University of Denmark, Kgs, Lyngby DK-2800, Denmark

**Keywords:** AOP, KER, Androgen receptor, Nipples, Reproductive toxicology, Endocrine disrupting chemicals

## Abstract

In rat developmental and reproductive toxicity studies, nipple/areola retention (NR) in male offspring is a biomarker for reduced androgen signaling during development. This is because nipples normally regress in male rats in response to androgen signaling during critical stages of development. NR is thus included as a mandatory endpoint in several OECD test guidelines for assessment of chemicals, particularly as a readout for anti-androgenic effects relevant for reproductive toxicity. With the growing interest in developing Adverse Outcome Pathways (AOPs) to aid in chemical risk assessment, a more pragmatic approach has been proposed, whereby essential units of knowledge could be developed independently of complete AOPs, not least emergent key event relationships (KERs). Herein, we have developed a KER linking “androgen receptor antagonism” and “increased areola/nipple retention”. The KER is based on a literature review conducted in a transparent semi-systematic manner in peer-reviewed databases with pre-defined inclusion criteria. Twenty-seven papers were included for development of the KER. The results support a qualitative relationship between the two key events (KEs) with a high weight of evidence; i.e., a causal relationship between androgen receptor (AR) antagonism and nipple retention in male rats exists.

## Highlights

• A full AOP KER description linking AR antagonism with nipple retention in rodents.
• Described KER 2133 is a non-adjacent KER of an intended AOP delineating anti-androgenicity as a mode for nipple retention.
• A case study for developing and publishing independent units of information under the AOP framework.

## Graphical abstract

**Figure:**
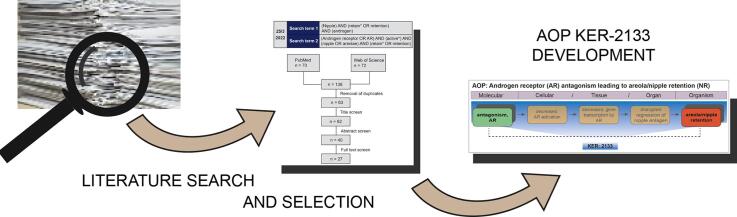

## Pretext

Adverse Outcome Pathways (AOPs) aim to depict causal toxicological pathways relevant for risk assessment, starting from a molecular initiating event (MIE) and culminating in an adverse outcome (AO) in an intact organism, or population. By providing mechanistic knowledge and links to measurable key events (KEs) that are essential for progressing through the causal pathway, the AOP framework can provide risk assessors with valuable information from which they can infer causality by using data from alternative test methods/assays. In essence, the unit that allows for inference is the key event relationship (KER) that links individual KEs along the causal pathway.

Since the development and peer-review of complete AOPs is very labor- and time-intensive, a more pragmatic approach to develop, review, and endorse AOPs using a more modular approach has been proposed (Svingen et al., 2021). This approach includes a formal recognition of emerging KERs as the core units of knowledge that could be developed and peer-reviewed independently of complete AOPs. As recently demonstrated, this could apply to both KERs for AOPs under development (Draskau et al., 2022, Panagiotou et al., 2022) or for incorporating new knowledge into existing AOPs (Huliganga et al., 2022). Herein, we have developed a KER linking androgen receptor (AR) antagonism and areola/nipple retention (NR) in reproductive toxicity studies.

## Introduction

Areola/nipple retention (NR) in male rat, or mouse, offspring is considered a biomarker for incomplete masculinization during fetal development. This is because male rats and mice normally do not display nipples, in contrast to female rats and mice that have 12 and 10 nipples, respectively (Imperato-McGinley et al., 1986, Mayer et al., 2008). This sexual dimorphism is believed to be largely due to differences in androgen signaling during development and thus NR in males can be considered a readout for compromised androgen action during critical developmental stages, as recently reviewed (Schwartz et al., 2021). Consequently, NR is included as a mandatory endpoint in several OECD test guidelines (OECD, 2008, OECD, 2013, OECD., 2018) on assessment of developmental and reproductive toxicity, not least to detect anti-androgenicity. As NR is measured *in vivo*, it is desirable to describe in detail the molecular and cellular mechanisms driving the effect, both qualitatively and quantitatively, in order to strengthen the predictive power of non-animal test data for chemical safety assessments.

Several mechanisms may lead to NR through reduced androgen signaling. These include disrupted steroidogenesis, inhibition of 5-α-reductase which will prevent conversion of testosterone to the more potent androgen receptor (AR) ligand dihydrotestosterone (DHT), and direct AR antagonism. These events may in themselves be both molecular initiating events (MIEs) or KEs depending on the chemical interactions with biomolecules. Thus, the ultimate goal for risk assessment purposes is to construct an AOP network that includes different MIEs or KEs (and AOPs) that, when affected, can lead to a common AO, in this case NR. Such AOP networks will aid the assessment of single chemical compounds, but also chemical mixtures, thus potentially accounting for cumulative effects in male reproductive toxicity (Christiansen et al., 2020, Conley et al., 2018, Conley et al., 2021, Howdeshell et al., 2017, Rider et al., 2010), or any other type of AO.

In the following, we have described the relationship between decreased androgen signaling by AR antagonisms and NR following AOP development principles (Developer’s Handbook available at https://aopwiki.org/). This is a non-adjacent KER enabling weight of evidence evaluation of studies that do not report on intermediate steps of the causal pathway; however, the overall aim is to eventually construct a robust AOP network for integrated assessment of anti-androgenic modalities leading to adverse outcomes. The modular descriptions will be openly available in AOP-wiki. The sections corresponding to entries in AOP-wiki are denoted with an asterisk (*) in the heading.

## Linking KER 2133 to an AOP

**Fig. 1:**
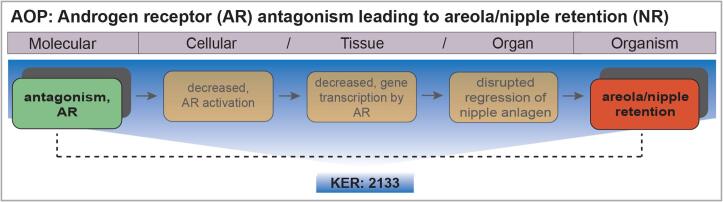
Schematic presentation of AOP 344 that is under development. The Key Event Relationship (KER) described in this report, KER 2133 (https://aopwiki.org/relationships/2133) connects the KE/MIE, “androgen receptor (AR) antagonism” and the KE/AO, “areola/nipple retention (NR)”.

The KER described in this report is part of an AOP that links AR antagonism with NR. The AOP identifier is 344 and it is available on AOP-wiki (https://aopwiki.org/aops/344). Fig. 1 shows a schematic presentation of the AOP in which the non-adjacent KER described in the present report is emphasized.

## Literature search strategy

**Fig. 2:**
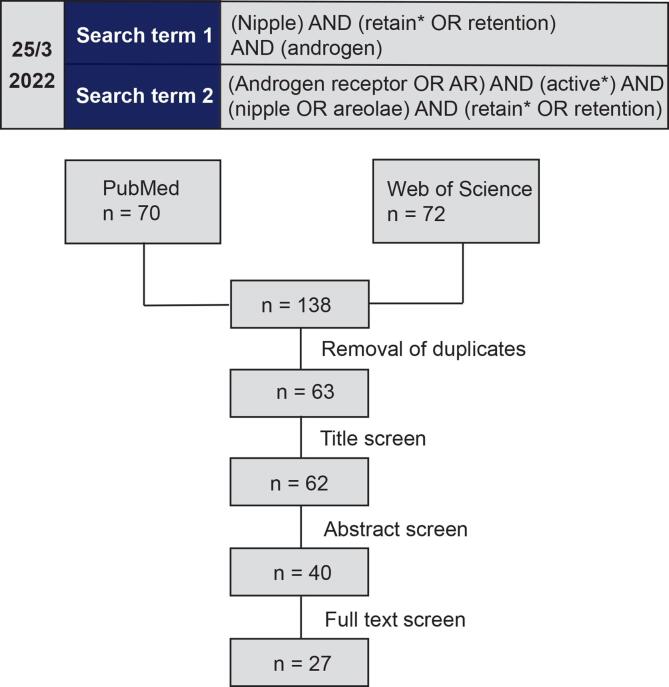
Workflow of the literature search strategy and the number of remaining papers after each screening step (title, abstract and full text, respectively). Papers were excluded based on pre-defined criteria.

A semi-systematic literature search was conducted during March 2022 in the peer-reviewed databases PubMed and Web of Science, using the search terms “(Nipple) AND (retain* OR retention) AND (androgen)” as well as “(Androgen receptor OR AR) AND (active*) AND (nipple OR areolae) AND (retain* OR retention)”. These searches resulted in 138 papers in total (Fig. 2). Upon removal of duplicates, papers were screened according to title, abstract and ultimately full text based on pre-defined inclusion criteria. *In vivo* studies were included if (i) the study was carried out in mice or rats, (ii) NR in males was investigated as an endpoint, (iii) AR antagonism was the suspected mechanism of action and (iv) anti-androgenic effects of single substance exposures (i.e., not studies on chemical mixtures) were investigated. *In vitro* studies were included if they contained mechanistic information on AR inhibition by chemical stressors.

## Key events linked by KER 2133

KER 2133 connects KE 26: AR antagonism with KE 1786: areola/nipple retention. This is a non-adjacent KER, as depicted in Fig. 1. The AOP-wiki entries for KE 26 and KE 1786 have been developed together with KER 2133 but not yet peer-reviewed. Hence, both KEs are included herein. NR as a KE draws on the extensive review by Schwartz et al. (2021). The AOP units described here will be made available and updated on AOP-wiki.

## KE 26: Antagonism, androgen receptor (*)

**Level of Biological Organization:** Molecular.

**Cell term:** Eukaryotic cells.

### Key event description

#### The androgen receptor (AR) and its function

Development of the male reproductive system and secondary male characteristics is dependent on androgens (foremost testosterone (T) and dihydrotestosterone (DHT). T and the more biologically active DHT act by binding to the AR (MacLean et al., 1993, MacLeod et al., 2010, Schwartz et al., 2019), with human AR mutations and mouse knock-out models having established its pivotal role in masculinization and spermatogenesis (Walters et al., 2010). The AR is a ligand-activated transcription factor belonging to the steroid hormone nuclear receptor family (Davey & Grossmann, 2016). The AR has three domains: the *N*-terminal domain, the DNA-binding domain, and the ligand-binding domain, with the latter being most evolutionary conserved. Apart from the essential role AR plays for male reproductive development and function (Walters et al., 2010), the AR is also expressed in many other tissues and organs such as bone, muscles, ovaries and the immune system (Rana et al., 2014).

#### AR antagonism as key event

The main function of the AR is to activate gene transcription in cells. Canonical signaling occurs by ligands (androgens) binding to AR in the cytoplasm which results in translocation to the cell nucleus, receptor dimerization and binding to specific regulatory DNA sequences (Heemers and Tindall, 2007). The gene targets regulated by AR activation depends on cell/tissue type and what stage of development activation occur, and is, for instance, dependent on available co-factors. Apart from the canonical signaling pathway, AR can also function through non-genomic modalities, for instance rapid change in cell function by ion transport changes (Heinlein & Chang, 2002). However, with regard to this specific KE the canonical signaling pathway is what is referred to.

### How it is measured or detected

AR antagonism can be measured *in vitro* by transient or stable transactivation assays to evaluate nuclear receptor activation. There is already a validated assay for AR (ant)agonism adopted by the OECD, Test No. 458: *Stably Transfected Human Androgen Receptor Transcriptional Activation Assay for Detection of Androgenic Agonist and Antagonist Activity of Chemicals* (OECD, 2020). The stably transfected AR-EcoScreen^TM^ cells (Satoh et al., 2004) should be used for the assay and is freely available for the Japanese Collection of Research Bioresources (JCRB) Cell Bank under reference number JCRB1328.

Other assays include the AR-CALUX reporter gene assay that is derived from human U2-OS cells stably transfected with the human AR and an AR responsive reporter gene (van der Burg et al., 2010), the MDA-kb2 cell line (Wilson et al 2004) and various other transiently transfected reporter cell lines (Körner et al., 2004), and more. Recently developed AR dimerization assay may soon be included in TGs for its improved ability to measure potential stressor-mediated dimerization/activation (Lee et al., 2021).

### Domain of application

#### Overview

Both the DNA-binding and ligand-binding domains of the AR are highly evolutionary conserved, whereas the transactivation domain show more divergence which may affect AR-mediated gene regulation across species (Davey & Grossmann, 2016). Despite certain inter-species differences, AR function mediated through gene expression is highly conserved, with mutations studies from both humans and rodents showing strong correlation for AR-dependent development and function (Walters et al., 2010).

#### Taxonomic application

Human, mouse, rat.

#### Life stages

Embryo (moderate), fetal (high), development through to adulthood (high).

### Stressors

- Cyproterone acetate: Using the AR-CALUX reporter assay in antagonism mode, cyproterone acetate showed an IC50 of 7.1 nM (Sonneveld et al., 2005).
- Epoxiconazole: Using transiently AR-transfected CHO cells, epoxiconazole showed a LOEC of 1.6 µM and an IC50 of 10 µM (Kjærstad et al., 2010).
- Flutamide: Using the AR-CALUX reporter assay in antagonism mode, flutamide showed an IC50 of 1.3 µM (Sonneveld et al., 2005).
- Flusilazole: Using hAR-EcoScreen Assay, triticonazole showed a LOEC for antagonisms of 0.8 µM and an IC50 of 2.8 (±0.1) µM (Draskau et al., 2019).
- Prochloraz: Using transiently AR-transfected CHO cells, prochloraz showed a LOEC of 6.3 µM and an IC50 of 13 µM (Kjærstad et al., 2010).
- Propiconazole: Using transiently AR-transfected CHO cells, propiconazole showed a LOEC of 12.5 µM and an IC50 of 18 µM (Kjærstad et al., 2010).
- Tebuconazole: Using transiently AR-transfected CHO cells, tebuconazole showed a LOEC of 3.1 µM and an IC50 of 8.1 µM (Kjærstad et al., 2010).
- Triticonazole: Using hAR-EcoScreen Assay, triticonazole showed a LOEC for antagonisms of 0.2 µM and an IC50 of 0.3 (±0.01) µM (Draskau et al., 2019).
- Vinclozolin: Using the AR-CALUX reporter assay in antagonism mode, vinclozolin showed an IC50of 1.0 µM (Sonneveld et al., 2005).

### Evidence for perturbation of this MIE by stressor

A large number of drugs and chemicals have been shown to antagonize the AR using various AR reporter gene assays. The AR is specifically targeted in AR-sensitive cancers, for example the use of the anti-androgenic drug flutamide in treating prostate cancer (Alapi & Fischer, 2006). Flutamide has also been used in several rodent *in vivo* studies showing anti-androgenic effects (feminization of male offspring) evident by e.g., short anogenital distance (AGD) in males (Foster and Harris, 2005, Hass et al., 2007, Kita et al., 2016). Quantitative Structure-Activity Relation (QSAR) models can predict AR antagonism for a wide range of chemicals, many of which have shown *in vitro* antagonistic potential (Vinggaard et al., 2008).

## KE 1786: Increase, nipple retention in male offspring (*)

**Level of Biological Organization:** Organism.

**Cell term:** Eukaryotic cells.

### Key event description

In common laboratory strains of rats and mice, females typically have 6 (rats) or 5 (mice) pairs of nipples along the bilateral milk lines. In contrast, male rats and mice do not have nipples. This is unlike e.g., humans where both sexes have 2 nipples (Schwartz et al., 2021).

In laboratory rats, high levels of dihydrotestosterone (DHT) induce regression of the nipples in males (Imperato-McGinley and Gautier, 1986, Kratochwil, 1977, Kratochwil and Schwartz, 1976). Females, in the absence of this DHT surge, retain their nipples. This relationship has also been shown in numerous rat studies with perinatal exposure to anti-androgenic chemicals (Schwartz et al., 2021). Hence, if juvenile male rats and mice possess nipples, it is considered a sign of perturbed androgen action early in life.

### How it is measured or detected

Nipple retention (NR) is visually assessed, ideally on postnatal day (PND) 12/13 (OECD., 2018, Schwartz et al., 2021). However, PND 14 is also an accepted stage of examination (OECD, 2013). Depending on animal strain, the time when nipples become visible can vary, but the assessment of NR in males should be conducted when nipples are visible in their female littermates (OECD, 2013).

Nipples are detected as dark spots (or shadows) called areolae, which resemble precursors to a nipple rather than a fully developed nipple. The dark area may or may not display a nipple bud (Hass et al., 2007). Areolae typically emerge along the milk lines of the male pups corresponding to where female pups display nipples. Fur growth may challenge detection of areolae after PND 14/15. Therefore, the NR assessment should be conducted prior to excessive fur growth. Ideally, all pups in a study are assessed on the same postnatal day to minimize variation due to maturation level (OECD, 2013).

NR is occasionally observed in controls. Hence, accurate assessment of NR in controls is needed to detect substance-induced effects on masculine development (Schwartz et al., 2021). It is recommended by the OECD guidance documents 43 and 151 to record NR as a quantitative number rather than a qualitative measure (present/absent or yes/no response). This allows for more nuanced analysis of results, e.g., high control values may be recognized (OECD, 2013, OECD., 2018). Studies reporting quantitative measures of NR are therefore considered stronger in terms of weight of evidence.

Reproducibility of NR results is challenged by the measure being a visual assessment prone to a degree of subjectivity. Thus, NR should be assessed and scored blinded to exposure groups and ideally be performed by the same person(s) within the same study.

### Biological domain of applicability

The applicability domain of NR is limited to male laboratory strains of rats and mice from birth to juvenile age.

### Regulatory significance of the adverse outcome

NR is recognized by the OECD as a relevant measure for anti-androgenic effects and is mandatory in the test guidelines Extended One Generation Reproductive Toxicity Study, TG 443 (OECD, 2018) and the two screening studies for reproductive toxicity, TGs 421/422 (OECD, 2016a, OECD, 2016b). The endpoint is also described in the guidance documents 43 (OECD, 2008) and 151 (OECD, 2013). Furthermore, NR data can be used in chemical risk assessment for setting the No Observed Adverse Effect Level (NOAEL) as stated in the OECD guidance document 151 (OECD, 2013): “*A statistically significant change in nipple retention should be evaluated similarly to an effect on AGD as both endpoints indicate an adverse effect of exposure and should be considered in setting a NOAEL*”.

### Stressors

An overview of chemical stressors causing nipple retention in male rats is provided in the main text and supplementary data (S1, Table 2) of Schwartz et al. (2021). The stressors and intrauterine exposure levels resulting in NR are listed below (Table 1).

**Table 2:** List of chemicals causing NR in male rat offspring (*in vivo*) due to exposure to an AR antagonist during development. Several of the chemical stressors have also been shown to antagonize AR *in vitro*; these are noted in the far-right column. Additional information, including species, strain, exposure period, time of NR measurement as well as the magnitude of NR at the effect dose is presented. * (p < 0.05). Based on semi-systematic literature review. Abbreviations: SD = Sprague-Dawley; LE = Long Evans; GD = Gestational Day; PD = Pup Day; LOAEL = Lowest Observed Adverse Effect Level; N.D. = Not determined. p,p’-DDE, dichlorodiphenyldichloro ethylene.

Species/Strain	Stressor	Exposure period	Time of measurement	NOAEL [mg/kg bw/day]	LOAEL [mg/kg bw/day]	Effect * p < 0.05		Reference
						Number of nipples	% nipples	
Rat/SD	Fenitrothion	GD 12–21	PD 13 *PD 100*	20 *N.D.*	25 *N.D.*	4.2 * (0)	–	Turner et al., 2002
Rat/SD	Flutamide	GD 6–PD 21	PD 14	N.D.	3.5	7 *	–	Schreiber et al., 2020
Rat/SD	Flutamide	GD 0–20	PD 56	2.5	10	3.37 ± 1.34 *	–	Lu et al., 2006
Rat/SD	Flutamide	GD 14–PD 3	PD 12	2.5	10	–	100	Miyata et al., 2002
Rat/SD	Flutamide	GD 12–21	PD 13 *PD 100*	N.D. *N.D.*	6.25 *N.D.*	10.2 * (8.3 *)	–	McIntyre et al., 2001
Rat/SD	Flutamide	GD 14–18	PD 13	N.D.	40	6 *	–	You et al., 1998
Rat/SD	Flutamide	GD 12–21	PD 14	N.D.	100	–	100	Mylchreest et al., 1999
Rat/Wistar	Flutamide	GD 6–PD 30	PD 12 *PD 20*	0.025 *N.D.*	0.25 *N.D.*	2.9 * (0)	–	Fussell et al., 2015
Rat/Wistar	Flutamide	GD 7–PD 16	PD 13	N.D.	0.77	2.8 *	–	Christiansen et al., 2008, Hass et al., 2007
Rat/LE	Flutamide	GD 14–18	PD 13	N.D.	40	6 *	–	You et al., 1998
Rat/SD	Linuron	GD 14–18	PD 13	N.D.	75	2.16 *	–	Hotchkiss et al., 2004
Rat/SD	Linuron	GD 12–21	PD 13 *PD 35* *PD 56*	N.D. *N.D.* *N.D.*	50 *N.D.* *N.D.*	3.3 ± 0.4 * (∼2 *) *N.D.*	–	McIntyre et al., 2002
Rat/SD	Linuron	GD 12–21	PD 13	25	50	3.7 *	–	McIntyre et al., 2000
Rat/SD	Linuron	GD 14–18	PD 10–13	N.D.	100	2.1 ± 0.7 *	–	Wolf et al., 1999
Rat/Wistar	Mancozeb	GD 7–PD 16	PD 13	6.25	25	0.6 ± 0.6 *	–	Hass et al., 2012
Rat/SD	p,p’-DDE	GD 14–18	PD 10–13	N.D.	100	3.13 ± 0.5 *	–	Wolf et al., 1999
Rat/SD	p,p’-DDE	GD 14–18	PD 13	N.D.	10	1.2 *	–	You et al., 1998
Rat/LE	p,p’-DDE	GD 14–18	PD 10–13	N.D.	100	0.74 ± 0.15 *	–	Wolf et al., 1999
Rat/LE	p,p’-DDE	GD 14–18	PD 13	10	100	3 *	–	You et al., 1998
Rat/Holtzman	p,p’-DDE	GD 14–18	PD 13	50	100	1.76 ± 0.56 *	–	Loeffler & Peterson, 1999
Rat/Wistar	Prochloraz	GD 6–PD 83	PD 12 *PD 20*	0.01 *N.D.*	5 *N.D.*	2.8 * (0)	–	Melching-Kollmuss et al., 2017
Rat/Wistar	Prochloraz	GD 7–PD 16	PD 13	8.75	35	1.7 ± 1.2 *	–	Hass et al., 2012
Rat/Wistar	Prochloraz	GD 7–PD 16	PD 13	25	30	3.6 [2.2;5.4] *	–	Christiansen et al., 2009
Rat/Wistar	Prochloraz	GD 7–PD 17	PD 13	N.D.	30	* (data not shown)	–	Vinggaard et al., 2006
Rat/Wistar	Procymidone	GD 7–PD 16	PD 13	N.D.	12.5	2.8 ± 1.2 *	–	Hass et al., 2012
Rat/Wistar	Procymidone	GD 7–PD 16	PD 13	N.D.	14.1	2.6 *	–	Christiansen et al., 2008, Hass et al., 2007
Rat/LE	Procymidone	GD 14–PD 3	PD 10–13	N.D.	100	3.75 ± 0.83 *	–	Wolf et al., 1999
Rat/SD	Pyrifluquinazon	GD 14–18	PD 13	12.5	25	4 *	–	Gray et al., 2019
Rat/Wistar	Tebuconazole	GD 7–PD 16	PD 13	12.5	50	1.6 ± 0.4 *	–	Hass et al., 2012
Rat/Wistar	Tebuconazole	GD 7–PD 16	PD 13	N.D.	50	3.43 ± 0.9 *	–	Taxvig et al., 2007
Rat/Wistar	Vinclozolin	GD 7–PD 16	PD 13	5	50	8.4 [6.9;9.6] *	–	Christiansen et al., 2009
Rat/Wistar	Vinclozolin	GD 7–PD 16	PD 13	N.D.	24.5	1.3 *	–	Christiansen et al., 2008, Hass et al., 2007
Rat /LE	Vinclozolin	GD 14–19	PD 13	N.D.	200	9.6 *	–	Wolf et al., 2000
Rat /LE	Vinclozolin	GD 14–15	PD 13	N.D.	400	4.86 ± 0.99 *	–	Wolf et al., 2000
Rat /LE	Vinclozolin	GD 16–17	PD 13	N.D.	400	8.84 ± 0.68 *	–	Wolf et al., 2000
Rat /LE	Vinclozolin	GD 17–PD 3	PD 13	12.5	50	–	100	(Ostby et al., 1999

**Table 1:** Example of chemical stressors, and exposure levels, leading to NR in male rat offspring. Information is adapted from Schwartz et al. (2021) and its supplementary material S1, Table 2.

Stressor	Exposure level [mg/kg bw/day]	References
BBP	500 – 750	Gray et al., 2000, Hotchkiss et al., 2004
DBP	100 – 642	Barlow et al., 2004, Carruthers and Foster, 2005, Clewell et al., 2013, Saillenfait et al., 2008, Wolf et al., 1999
DEHP	375 – 1500	Christiansen et al., 2010, Gray et al., 2000, Jarfelt et al., 2005, Moore et al., 2001, Saillenfait et al., 2009, Wolf et al., 1999
DiBP	125 – 625	Saillenfait et al., 2008
DiNP	750	Gray et al., 2000
DnHP	50 – 500	Saillenfait et al., 2009
Finasteride	0.01 – 320	Bowman et al., 2003, Imperato-McGinley et al., 1992, Martínez et al., 2011
Flutamide	0.0025 – 100	Foster and Harris, 2005, Fussell et al., 2015, McIntyre et al., 2001, Miyata et al., 2002
Linuron	50 – 100	Hotchkiss et al., 2004, Mcintyre et al., 2002, Wolf et al., 1999
p,p’-DDE	500	Wolf et al., 1999, You et al., 1998
Prochloraz	31.25 – 250	Melching-Kollmuss et al., 2017, Noriega et al., 2005, Vinggaard et al., 2005
Procymidone	25 – 200	Hass et al., 2007, Ostby et al., 1999, Wolf et al., 1999
Vinclozolin	1 – 400	Hellwig et al., 2000, Ostby et al., 1999, Schneider et al., 2011, Wolf et al., 2000, Wolf et al., 2004

BBP, butyl benzyl phthalate; DBP, di-butyl phthalate; DEHP, di-ethylhexyl phthalate; DiBP, di-isobutyl phthalate; DiNP, di-isononyl phthalate; DnHP, di-*n*-hexyl phthalate; p,p’-DDE, dichlorodiphenyldichloro ethylene.

## KER 2133: Androgen receptor (AR) antagonism leading to nipple retention (NR) in male offspring (*)

### Biological domain of applicability

Taxonomic applicability: Rats and mice.

Life stage applicability: Developmental.

Sex applicability: Male.

### KER description

Several chemicals can antagonize the androgen receptor (AR) *in vitro*, resulting in decreased AR activation. Decreased AR activation can lead to incomplete reproductive development in males, which can be expressed in several ways. One endpoint affected is areola/nipple retention (NR), which *in vivo* studies have shown to be linked to suppressed AR activation. NR in rat and mouse toxicity studies is considered an adverse effect (i.e., an AO).

### Evidence supporting this KER

#### Biological plausibility

The biological plausibility of a link between decreased AR activation and increased NR in male rats is high. The relationship is supported by numerous studies showing that several potent AR antagonists *in vitro* induce NR *in vivo*. However, in the literature review conducted for this KER, no studies in mice were found to fulfill the inclusion criteria. The present KER is hence exclusively a description of the situation in rats, although it is believed that the link also exists in mice.

The AR is activated through binding of either testosterone or dihydrotestosterone (DHT), the latter having the highest affinity for the AR. Upon binding, the AR translocates to the target cell nucleus where it acts as a transcription factor (Albert, 2018).

NR has been shown to be more dependent on DHT-signaling, which suggests that chemicals inducing increased NR also have a higher affinity for the AR than DHT in order to outcompete DHT for AR binding, although supra-high doses of chemicals with lower AR affinity could be speculated to also outcompete T or DHT. The general principle of higher affinity, however, has been confirmed by *in vitro* studies (Gray et al., 2019, Hass et al., 2012, McIntyre et al., 2000).

#### Empirical evidence

Table 2 lists chemical stressors shown to antagonize the AR *in vitro* as well as causing NR in male rat offspring *in vivo.* Additional information from the *in vivo* studies, including the animal species and strain, as well as the doses tested, the dosing period and the time of measurement of NR are specified in this table. The lowest dose yielding a significant increase of retained nipples in male rat pups is defined as the LOAEL. Conversely, the NOAEL represents the highest tested dose yielding no significant increase in NR. Note that the given NOAEL and LOAEL values are highly dependent on study design. Significant values are marked with an asterisk.

Table 3 shows a list of stressors shown to have AR antagonistic properties *in vitro* or in other *in vivo* studies, but for which the doses tested *in vivo* did not produce a significant effect on NR. In this list, the lowest tested dose is reported, and the NOAEL presents the highest dose tested which produced no statistically significant effect on NR. Apart from the NOAEL, the information given in Table 3 is identical to Table 2.

**Table 3:** List of chemicals that caused no significant effect on NR *in vivo* despite being known to have AR antagonistic properties in *in vitro* studies or previous *in vivo* experiments. The highest dose tested that led to no significant effect is presented as the NOAEL. Additional information, including species, strain, exposure period, time of NR measurement as well as the magnitude of NR at the NOAEL is presented. Based on semi-systematic literature review. Abbreviations: SD = Sprague-Dawley; GD = Gestational Day; PD = Pup Day; NOAEL = No Observed Adverse Effect Level. p,p’-DDE, dichlorodiphenyldichloro ethylene.

Species/Strain	Stressor	Exposure period	Time of measure-ment	Lowest dose tested	NOAEL [mg/kg bw/day]	Effect (number of nipples)	Reference
Rat/SD	Bisphenol C	GD 14–18	PD 13	12.5	200	1.21 (NS)	Gray et al., 2019
Rat/SD	p,p’-DDE	GD 6–PD 20	PD 13	5	50	NS *(data not shown)*	Yamasaki et al., 2009
Rat/Wistar	Epoxiconazole	GD 7–PD 16	PD 13	3.75	15	0.5 ± 1.0 (NS)	Hass et al., 2012
Rat/Wistar	Epoxiconazole	GD 7–PD 16	PD 13	15	50	3.38 (NS) a	Taxvig et al., 2007
Rat/SD	Linuron	GD 6–PD 21	PD 14	1.5	12.5	0.6 (NS)	Schreiber et al., 2020
Rat/SD	Fenitrothion	GD 1–PD 21	PD 12	Gestation: 0.62 Lactation: 1.32	Gestation: 3.75 Lactation: 7.75	0.0 ± 0.0	Okahashi et al., 2005

a This study had a control group with NR = 2.08, which can explain the non-significance compared to the exposure group despite the high NR value.

#### Uncertainties

A major challenge with NR as a biomarker is the subjectivity of the measure. In juvenile rat pups, nipples are only present as areolae, i.e., dark shadows with or without a nipple bud. This means that the experience of the personnel assessing the presence and number of areolae/nipples can influence the results. Furthermore, the results are likely prone to larger variation if several assessors are used to record NR within the same study. To minimize these sources of uncertainty, assessors must be trained to recognize areolae and not look for fully developed nipples. Moreover, the number of assessors should be limited to one or two, and they should always be blinded to exposure groups.

Another factor that may affect NR results is the age of the rat pups at the time of assessment. OECD guidelines have standardized the time for measuring occurrence of NR to be optimal at PD 12 or 13, when they are visible in female littermates (OECD, 2013). However, assessment of permanent NR is not included in any international guidelines. Hence, if NR is measured in older offspring, the time of measurement is not consistent between studies and varies between PD 20 and PD 100. Thus, conclusions on whether NR is permanent or not may differ based on study design. This distinction between a transient and a permanent effect is important from a regulatory perspective, since only a permanent effect will be categorized as a malformation according to OECD guidance document 43 (OECD, 2008).

### Quantitative understanding

The quantitative understanding of the relationship between decreased AR activity and NR is challenged by the fact that the potency of AR antagonism *in vitro* is not proportional to the magnitude of NR observed *in vivo* (Gray et al., 2019). Hence, predicting *in vivo* effects based on *in vitro* data is not yet possible. However, *in vitro* studies can give indications of which chemicals might exhibit anti-androgenic effects *in vivo* and should be subject to further testing (Hass et al., 2012). Development of more representative *in vitro* models is necessary if *in vivo* tests are to be phased out entirely.

### Timescale

NR manifest in juvenile male rat pups in response to reduced androgen signaling, e.g. resulting from exposure to an anti-androgenic chemical stressor during fetal life. Developmental sensitivity during fetal development is highest during the so-called male masculinization programming window (MPW) which in rats is between gestational day (GD) 15 and 19 (Welsh et al., 2008).

A study in which pregnant rat dams were exposed to the AR antagonist vinclozolin for two-day periods during gestation showed that GD 16–17 was the most sensitive period for increased NR in male offspring (Wolf et al., 2000). A similar study using di-*n*-butyl phthalate (reduces testosterone levels) also showed that GD 16–17 was the most sensitive period for increased NR in male rats (Carruthers & Foster, 2005). However, to determine if other chemical stressors also have the highest antagonistic potential towards the AR during GD 16–17, further studies with a similar design would be informative.

NR can only be recorded when pups are old enough to display them, yet before excessive fur has developed. Hence, the most accurate results can be obtained from assessing the number of nipples on PD 12–14 depending on rat strain and the time of female littermates displaying nipples (OECD, 2013).

### Known modulating factors

One factor that may influence NR counts in toxicity studies is the rat strain. In the studies included for development of the present KER, Wistar and Sprague-Dawley rats are the most widely used. Additionally, some studies have reported effects in Long Evans hooded rats and Holtzman rats.

An extensive review on NR effects reports no major differences on the magnitude of effect between Sprague-Dawley and Wistar rats (Schwartz et al., 2021). Thus, results from the two rat strains appear comparable. However, attention should be paid when comparing of NR between Sprague-Dawley and Long Evans hooded rats. For example, when exposed to flutamide or p,p’-DDE during GD 14–18, the Sprague-Dawley and Long Evans strains are equally sensitive to flutamide exposure, but Sprague-Dawley rats are more sensitive towards exposure to p,p’-DDE (You et al., 1998). The LOAEL for p,p’-DDE exposure in Sprague-Dawley rats was estimated to be 10-fold lower than in Long Evans rats. This finding is supported by another study showing that Sprague-Dawley rats present 4 times as many nipples as Long Evans hooded rats when exposed to 100 mg/kg bw/day p,p’-DDE during GD 14–18 (Wolf et al., 1999).

### Response-response relationship

No response-response relationship has been identified.

### Known feedback loops influencing this KER

No feedback loops that could influence the KER have been identified.

### Classification of quantitative understanding

The quantitative understanding of the present KER remains low.

## Conclusion

We conclude that a causal relationship exists between the KEs “androgen receptor (AR) antagonism” and “areola/nipple retention” in male rat offspring. These are non-adjacent KEs essentially linking a MIE with an AO. Based on a semi-systematic review of available literature, evidence for this qualitative relationship is strong. Methods for reliably quantifying how much the AR must be inhibited before significant NR manifest remains insufficient. Available *in vivo* studies report NR with a high degree of variability, which can be attributed to different sources of uncertainty. It is hence not possible to isolate and correct for confounding factors at present. Further studies following OECD guidelines and, ideally, conducting both *in vitro* and *in vivo* assays are necessary before a general model can be developed and applied for predictive toxicology with quantitative power. OECD test guidelines relevant to AOP 344 include *in vitro* and *in vivo* detection of AR antagonism (TG 458 and 441, respectively) and rodent reproductivity toxicity studies assessing NR (TG 443; TG 421/422) (OECD, 2009, OECD, 2016a, OECD, 2016b, OECD., 2018, Oecd, 2020).

## Funding

This work was funded by grants from The Danish Veterinary and Food Administration (DVFA), Project “Feminix”, and The Nordic Working Group on Chemicals, Environment and Health (NKE) sub-group Nord UTTE (project 2020–027 and 2021–020).

## CRediT authorship contribution statement

**Emilie Bak Pedersen:** Methodology, Investigation, Data curation, Writing – original draft, Writing – review & editing. **Sofie Christiansen:** Conceptualization, Supervision, Investigation, Data curation, Writing – review & editing, Funding acquisition. **Terje Svingen:** Methodology, Investigation, Data curation, Supervision, Writing – original draft, Writing – review & editing, Funding acquisition.

## Declaration of Competing Interest

The authors declare that they have no known competing financial interests or personal relationships that could have appeared to influence the work reported in this paper.
